# Associations Between Psychological Factors and Adherence to Health Behaviors After Percutaneous Coronary Intervention: The Role of Cardiac Rehabilitation

**DOI:** 10.1093/abm/kaae008

**Published:** 2024-03-02

**Authors:** Emma R Douma, Willem J Kop, Nina Kupper

**Affiliations:** Department of Medical and Clinical Psychology, Center of Research on Psychological Disorders and Somatic Diseases (CoRPS), Tilburg University, Tilburg, The Netherlands; Department of Medical and Clinical Psychology, Center of Research on Psychological Disorders and Somatic Diseases (CoRPS), Tilburg University, Tilburg, The Netherlands; Department of Medical and Clinical Psychology, Center of Research on Psychological Disorders and Somatic Diseases (CoRPS), Tilburg University, Tilburg, The Netherlands

**Keywords:** Coronary heart disease, Health behavior change, Cardiovascular rehabilitation, Percutaneous coronary intervention, Psychological factors

## Abstract

**Background:**

Cardiac rehabilitation (CR) participation after percutaneous coronary intervention (PCI) for coronary heart disease lowers the disease burden and risk of recurrent cardiac events. Examining psychological factors may improve post-PCI health behavior adherence.

**Purpose:**

To determine whether psychological factors are associated with post-PCI health behavior adherence, and the role of CR participation.

**Methods:**

Data from 1,682 patients (22.1% female, *M*_age_ = 64.0, *SD*_age_ = 10.5 years) from the THORESCI cohort were included. Adjusted mixed models were used to examine associations between psychological factors and the 1-year course of health behaviors, using interactions to test for moderation by CR participation.

**Results:**

Psychological factors were associated with the trajectories of adherence to medical advice, exercise, and diet. The strongest association found was between optimism and the trajectory of dietary adherence (*B*: = −0.09, *p* = .026). Patients with high optimism levels had a worse trajectory of dietary adherence compared to patients with low to middle optimism levels. Participation in CR buffered the associations of high anxiety, pessimism, and low to middle resilience, but strengthened the associations of high stress in the past year with the probability of smoking.

**Conclusions:**

Psychological factors are associated with post-PCI health behavior adherence, but the pattern of associations is complex. Patients with high levels of anxiety, pessimism, and low to middle resilience levels may disproportionately benefit from CR. Cardiac rehabilitation programs could consider this to improve post-PCI health behavior adherence.

**Clinical Trials Registration #:**

NCT02621216.

## Teaser

Psychological characteristics of patients with coronary heart disease are associated with health behavior adherence and patients with high levels of anxiety, pessimism, and low to middle resilience levels may disproportionately benefit from cardiac rehabilitation.

## Introduction

Cardiovascular diseases are the leading cause of death globally, with coronary heart disease (CHD) contributing most to this high burden [1]. State-of-the-art treatment options, such as coronary revascularization through percutaneous coronary intervention (PCI) and long-term antiplatelet medications have contributed to lowered mortality rates [2–4]. As a result, the clinical management of CHD has become more chronic in nature and requires comprehensive disease management through control of behavioral risk factors and improvement of quality of life, both of which are targeted through cardiac rehabilitation (CR) [5]. Evidence indicates that CR is a cost-effective strategy that can result in a lower disease burden, reduced risk of recurrent cardiac events, and improved quality of life [3].

An important component of CR is assisting patients with adherence to medical advice related to cardiovascular health and with adherence to cardiovascular risk-reducing behaviors. Relevant health behaviors include sufficient physical activity, medication adherence, eating a cardiac-healthy diet, reducing stress, and smoking cessation [3, 5]. Previous research has demonstrated that adherence to these health behaviors (meaning both adherence to medical advice and adherence to risk-reducing health behaviors) can protect against future cardiovascular events and death [6–8]. Specifically, adherence to more than one health behavior has been found to be associated with a 66% reduced risk of overall cardiovascular disease compared to adopting none or only one health behavior [9]. Despite these benefits, research has also shown that the uptake of health behaviors and subsequent long-term adherence is suboptimal, as cardiac patients often relapse into former adverse health behavior habits or dropout during the course of CR [10, 11].

**Fig. 1.**
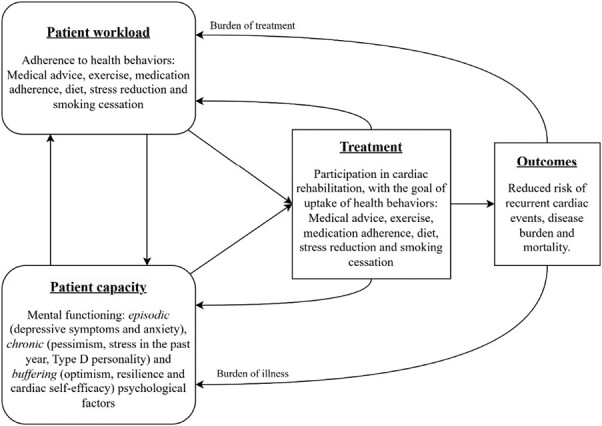
Model of patient complexity, adapted from Shippee et al. [12].

Following Shippee’s patient-centered model of patient complexity (Fig. 1) [12], to generate positive outcomes for patients, it is important to ensure *patient workload* is balanced with *patient capacity*. Long-term adherence to health behavior can be characterized as part of the *patient workload*, positive and negative psychological characteristics as part of the *patient capacity,* and participation in CR as *treatment*. This complex interaction influences *outcomes* (reduced risk of recurrent cardiac events, disease burden, and mortality), which in turn influences a patient’s *burden of treatment*, which feeds back into patient workload, and *burden of illness*, which feeds back into patient capacity. An imbalance between patient workload and capacity may be linked to negative outcomes. Therefore, investigating the association between capacity (psychological factors) and workload (adherence to health behaviors), as well as the role of treatment (participation in CR), may contribute to a better understanding of a balanced workload and capacity, and improve outcomes for this patient population.

Previous research on the uptake of health behaviors in patients with CHD has identified practical and motivational barriers, such as unwillingness to adopt a new lifestyle, lacking time, or lacking knowledge [13, 14], all of which lower a patient’s capacity for taking up health behaviors. In addition, several negative episodic psychological factors, particularly *depression* and *anxiety*, as well as chronic psychological factors, such as *pessimism, stress in the past year*, and *Type D personality* have been shown to contribute to adverse lifestyle behaviors in patients with CHD or similar populations [15–19]. For example, pessimism has been hypothesized to contribute to CHD via unhealthy dietary behaviors [20]. Type D *(Distressed)* personality, the tendency to experience a high joint occurrence of negative affectivity (NA) and social inhibition (SI) [21], has been related to a lower uptake of exercise behavior, less healthy eating behaviors, and worse treatment adherence [19].

In addition to these negative psychological constructs that are associated with a reduced patient capacity for health behavior uptake, buffering (positive) psychological factors have been associated with health behavior uptake as well. For example, *optimism*, *resilience*, and *cardiac self-efficacy* have been shown to be associated with healthier behaviors, but their importance varies with the associated behavior [22–24]. Optimism, cardiac self-efficacy, and resilience have been related to physical activity and healthy dietary habits [22, 25, 26], whereas resilience has additionally been related to smoking cessation [23]. These psychological factors have mainly been related to the *uptake* of health behaviors through correlational research, but longitudinal research on *adherence* to health behaviors is sparse.

The goal of the current study is therefore to explore whether chronic, episodic, and buffering psychological factors are associated with long-term adherence to specific health behaviors among patients with CHD following PCI. This study focuses on psychological factors that have been associated with the risk of CHD outcomes in prior research. Furthermore, it is important to investigate whether participation in CR buffers or strengthens any of these associations, to inform future development and application of CR and balance the burden of treatment and burden of illness for patients with CHD. Based on recommendations for CR from the European Society of Cardiology, the specific health behaviors under investigation are: following medical advice, sufficient physical activity, medication adherence, eating a cardiac-healthy diet, reducing stress, and smoking cessation [24].

## Methods

### Participants and Procedure

The current study uses data from the “Tilburg Health Outcomes Registry of Emotional Stress after Coronary Intervention (THORESCI)” study [27]. This is an ongoing observational cohort study on the prevalence and predictive value of psychological factors in patients undergoing PCI [PMID: 27922567]. Participants were recruited from the cardiology unit of the Elisabeth-TweeSteden Hospital (ETZ) in Tilburg, The Netherlands at the time of their hospitalization for PCI. Patients who underwent either elective or acute PCI, who were over 18 years of age, and had a sufficient understanding of the Dutch language were eligible for inclusion. Patients were ineligible to participate if they suffered from life-threatening comorbidities (e.g., metastasized cancer), language barriers, cognitive disorders (i.e., vascular dementia or Alzheimer’s disease), and/or a disability that prevented them from filling out questionnaires (e.g., blindness).

A member of the research team approached patients on the day of the PCI to explain the study. When patients agreed to participate, they signed an informed consent form and were asked to fill out validated questionnaires at several time points post-PCI. The baseline included two surveys, at 5 days and 30 days post-PCI. For the current study, we used 5-day post-PCI survey data and if those data were not present, the 30-day post-PCI survey data were used. All personality questionnaires were assessed at 30 days post-PCI. Mood questionnaires were assessed at both time points. Follow-up questionnaires were conducted at 6 and 12 months post-PCI. Data used in this study were collected between December 2013 and February 2021. The Coronavirus disease 2019 (COVID-19) pandemic negatively affected inclusion between March 2020 and May 2021, as the hospital did not allow researchers on the cardiology and coronary care unit, because of the COVID-19-related social restrictions. Ethics approval was obtained from the local medical ethics review board (METC Brabant protocol number: *NL46259.028.13*).

### Materials

#### Adherence to medical advice and health behaviors

Self-reported treatment adherence was assessed using the Medical Outcomes Study, part 1 (MOS) [28]. Participants indicated on a 6-point Likert scale ranging from 1 (never) to 6 (always) how often they complied with general medical treatment. An example statement is “I had a hard time doing what the doctor told me to do.” The total score ranges from 5 to 30, with higher scores indicating a higher level of adherence to medical advice. For this study, scores on the MOS subscales relating to adherence to medical advice, exercise adherence, dietary adherence, and stress reduction were used. Patients indicated how often they complied with the behavior in the last 4 weeks on a 6-point Likert scale ranging from 1 (never) to 6 (always). An example question for physical activity is “How often in the past 4 weeks have you exercised regularly?”

To measure medication adherence, three questions were used. One question from the Heart & Soul study (“How often did you take your medication according to the prescription?”) [29, 30], and two questions related to two known barriers of medication adherence (forgetting and special reasons not to take medications) were used [31]. Participants were asked to answer on a 5-point Likert scale ranging from 0 (never) to 4 (all the time). An unpublished exploratory factor analysis on a random 50% sample of the dataset demonstrated these three items to load on 1 factor, together explaining 68% of the variance. Internal consistency analysis in R (McDonald’s omega) on another random 50% sample showed a McDonald’s omega of .90, with factor loadings of .92 (item 2: regime), .78 (item 3: special reasons), and .87 (item 1: forgetting). A total score was therefore constructed that ranged from 0 to 12 with higher scores indicating worse medication adherence.

Smoking was assessed through a purpose-designed question asking patients whether they smoked. Patients could answer, “yes,” “no,” or “quit.” The responses “no” and “quit” were collapsed into one category, as measuring current behavior was the objective of this study.

#### Psychological factors

##### Depressive symptoms.

The Patient Health Questionnaire (PHQ-9) was used to measure symptoms of depression at baseline [32]. Participants indicated on a 4-point Likert scale ranging from 0 (not at all) to 3 (nearly every day) to what extent they experienced depressive symptoms. An example question is: “(Are you) feeling bad about yourself—or that you are a failure or have let yourself or your family down?” The total PHQ-9 score ranges between 0 and 27 with a higher score indicating higher depression severity. Cronbach’s α in the current sample = .89.

##### Anxiety.

The level of anxiety was measured with the General Anxiety Disorder questionnaire (GAD-7) at baseline [33]. Participants indicated on a 4-point Likert scale ranging from 0 (not at all) to 3 (nearly every day) to what extend they experienced certain feelings reflecting anxiety. An example question is: “Over the last two weeks, how often have you been bothered by the following problems: feeling nervous, anxious or on edge?” The total score ranges from 0 to 21 with a higher score indicating higher feelings of anxiety. Cronbach’s α in the current sample = .92.

##### Optimism and pessimism.

Optimism and pessimism were assessed using the 10-item Life Orientation Test—Revised (LOT-R) questionnaire at baseline [34]. Participants indicated on a 5-point Likert scale ranging from 0 (strongly disagree) to 4 (strongly agree) to what extent they agreed with statements pertaining to optimism or pessimism. An example statement for optimism is: “In uncertain times, I usually expect the best.” For pessimism, an example statement is: “If something can go wrong for me, it will.” Of the 10 items, 3 assess optimism and 3 assess pessimism. The remaining four items are considered filler items and do not contribute to the total score. The total score on both subscales ranges between 0 and 12, with a higher score indicating higher levels of optimism or pessimism. For the current study, separate total scores were calculated for optimism and pessimism. Prior research has demonstrated that optimism and pessimism are two separate constructs that can both be measured using the LOT-R [34]. Cronbach’s α in the current sample = .70 for optimism and .72 for pessimism.

##### Resilience.

The Dispositional Resilience Scale (DRS-15) was administered at baseline to determine stress resilience and included cognitive, emotional, and behavioral qualities [35]. Positively and negatively worded items are rated on a 4-point Likert scale from 0 (“not at all true”) to 3 (“completely true”). An example statement is: “I enjoy the challenge when I have to do more than one thing at a time.” The total score ranges between 0 and 45, with a higher score indicating higher resilience. Cronbach’s α in the current sample = .78.

##### Stress in the past year.

Participants were asked at baseline to indicate their experienced stress over the past year on a 4-point Likert scale ranging from 1 (almost no stress at all) to 4 (a lot of stress) [36].

##### Type D personality.

Type D personality was measured using the Type D scale-14 (DS14) at baseline [21], which includes the assessment of two stable personality traits: negative affectivity (NA) and social inhibition (SI). Participants are asked to rate items on a 5-point Likert scale ranging from 0 (false) to 4 (true). A separate score was calculated for both subscales of Type D personality. A continuous method was used to assess Type D personality, meaning the main effects of NA and SI, their quadratic effects (NA*NA and SI*SI) as well as their interaction (NA*SI) were added to each model that included type D personality [37]. As only NA*SI (Type D personality) falls within the scope of the current study, the main and quadratic effects of NA and SI are reported in the Supplementary materials (Supplementary Table 1 and Supplementary Figs. 1–7). Cronbach’s α in the current sample = .90 for NA and .89 for SI.

##### Cardiac self-efficacy.

Cardiac self-efficacy was measured at baseline using a Dutch version of the Cardiac Self-efficacy Scale (CSE scale) [38]. Participants indicated on a 5-point Likert scale ranging from 1 (not at all confident) to 5 (completely confident) to what extent they feel confident regarding each of the statements. An example statement is “How confident are you that you can get regular aerobic exercise (work up a sweat and increase your heart rate)?” Total scores range from 16 to 80, with a higher score indicating more cardiac self-efficacy. Cronbach’s α in the current sample = .91.

#### Sociodemographic and medical characteristics

##### Sociodemographic variables.

Sociodemographic variables were obtained from the baseline survey and included age, biological sex, level of education (stratified as low: high school or lower, or high: all other levels of education), and whether participants lived with a spouse or partner.

##### Cardiac rehabilitation.

Patients indicated whether they participated in CR in any hospital or outpatient clinic by answering either “yes” or “no” in the survey at 30 days and in the survey at 6 months. For participants who received CR in the ETZ hospital this self-report measure was verified by review of the medical records. Five percent of ETZ CR participants indicated to have missed at least half of the CR sessions (4 out of 8 or more).

##### Medical history.

Baseline medical data were extracted from electronic medical records and included information on the PCI procedure (elective vs. acute), presence of familial history of heart disease (genetic risk, based on a positive family history of cardiovascular disease onset <60 years), previous cardiovascular events, or procedures (heart failure, myocardial infarction [MI], cerebrovascular accident [CVA]/transient ischemic attack [TIA], previous coronary procedures (i.e., bypass grafting [CABG], PCI)), cardiovascular risk factors (i.e., hypertension, obesity [BMI ≥ 30 kg/m^2^]), and comorbidities (i.e., diabetes mellitus, non-metastasized malignancy, kidney disorder).

### Statistical Analysis

All data were analyzed using SPSS version 28 [39]. Baseline characteristics are presented as mean ± standard deviation (*SD*) for continuous variables or as *N* (%) for categorical data. Differences in health behaviors at baseline between patients who participated in CR and patients who did not participate in CR were inspected using independent sample *t*-tests for adherence to medical advice, exercise adherence, medication non-adherence, dietary adherence, and stress reduction and a chi-squared test for smoking, to ensure potentially identified differences between these groups in terms of adherence arose over time. The absence of multicollinearity was confirmed using Pearson’s correlations between all psychological factors.

Repeated linear mixed models were used to explore longitudinal changes and associations of adherence to medical advice, exercise adherence, medication non-adherence, dietary adherence, and stress reduction with the psychological predictors. Repeated logistic mixed models were used for smoking. The large sample size (*N* = 1,682) enabled the evaluation of all psychological predictors in one multivariable model (i.e., depressive symptoms, anxiety, pessimism, stress in the past year, Type D personality, optimism, resilience, and cardiac self-efficacy) as well as participation in CR. Repeated linear and logistic mixed models calculate estimates with 95% confidence intervals and account for missing data and within-subject correlations. For each health behavior, four separate models were constructed. Model 1 included the independent variable time, reflecting longitudinal changes, and adherence to medical advice or the separate health behaviors as dependent variables. The between-subject effects of psychological predictors at baseline on adherence to medical advice or adherence to health behaviors were included in model 2. The within-subject effects of psychological predictors at baseline on the course of adherence to medical advice or health behaviors were inspected by including interactions between time and the psychological predictors in model 3. In model 4, the three-way interactions between participation in CR, psychological predictors at baseline and time were added to the model to test whether CR participation moderated any of the associations between psychological factors and adherence to medical advice or health behaviors. Models 2, 3, and 4 were adjusted for sociodemographic and medical characteristics: age, sex, educational level, civil status, previous cardiovascular events or procedures, comorbidities, genetic risk, PCI indication (elective vs. acute), obesity, and hypertension. A covariance pattern model was fitted to the data in linear and logistic mixed models. The dependencies in the repeated measures were modeled with an unstructured covariance matrix. To test whether more economical covariance matrices would better fit the data, likelihood ratio tests were used. Significance levels for the generalized linear model were tested using the Satterthwaite approximation.

To facilitate the interpretation of interaction effects, groups were created using cut-off points based on the mean, mean + *SD*, and mean − *SD* values of psychological variables. For non-normally distributed variables, three equal groups were created. Cut-off points for depressive symptoms and anxiety have previously been determined and were used to create groups for these psychological variables [32, 33]. To visualize interaction terms, estimated marginal means (EMM) of the score on adherence were plotted over time for the three groups. To visualize three-way interactions, EMM were plotted separately for patients who participated in CR and for patients who did not. To visualize interaction terms for smoking, the predicted values from the respective models were used.

## Results

### Participant Characteristics

Table 1 shows the baseline characteristics of the 1,682 patients included in the analyses. On average, patients were 64.0 (*SD* = 10.5) years old, 22% were female, and most patients had a high education level (61%). Most patients (68%) had received acute PCI treatment. A quarter of the patients (25%) had one or more comorbidities, whereas 41% of the patients had previously experienced a cardiovascular event or procedure. Based on a BMI cut-off of ≥ 30 kg/m^2^, 29% of the patients had obesity. A total of 1,132 (67%) patients participated in CR, with 5% missing at least four appointments. At baseline, no differences were found in adherence to medical advice or adherence to health behaviors between patients who participated in CR and those who did not.

**Table 1:** Patient Characteristics [Reported as Mean ± *SD* or *N* (%)]

	Total (*N* = 1,682)	CR participation (*N* = 1,132)	No CR participation (*N* = 535)
Age in years (range)	64 ± 10.5 (23–95)	62.4 ± 10.1	67.3 ± 10.6
Sex (female)	371 (22.1%)	261 (23.1%)	106 (19.8%)
Partner (yes)	1,356 (80.6%)	925 (81.8%)	421 (78.7%)
Education			
High	515 (30.6%)	328 (31.5%)	183 (37.1%)
Low	1,026 (61%)	713 (68.5%)	310 (62.9%)
PCI elective	534 (31.7%)	326 (28.9%)	204 (38.2%)
PCI acute	1,144 (68.0%)	803 (71.1%)	330 (61.8%)
Familial history of CVD	660 (39.2%)	483 (42.7%)	170 (31.8%)
Obesity (BMI ≥ 30)	493 (29.3%)	331 (29.2%)	158 (29.5%)
Hypertension	746 (44.4%)	471 (41.6%)	268 (50.1%)
Previous CVD (event or procedure)	697 (41.4%)	425 (37.5%)	266 (49.7%)
MI	385 (22.9%)	232 (20.5%)	149 (27.9%)
CVA	79 (4.7%)	48 (4.2%)	29 (5.4%)
TIA	31 (1.8%)	12 (1.1%)	19 (3.6%)
CABG	127 (7.6%)	71 (6.3%)	56 (10.5%)
PCI	522 (31%)	316 (27.9%)	201 (37.6%)
Heart failure	59 (3.5%)	40 (3.5%)	19 (3.6%)
Comorbidities	416 (24.7%)	271 (23.9%)	142 (26.5%)
Diabetes mellitus	324 (19.3%)	213 (18.8%)	109 (20.4%)
Malignancy, excluding metastasized cancer	81 (4.8%)	50 (4.4%)	30 (5.6%)
Kidney disorder	59 (3.5%)	34 (3%)	25 (2.7%)

### Adherence Over Time

**Fig. 2.**
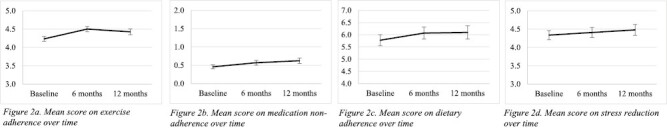
(a) Mean score on exercise adherence over time. (b) Mean score on medication non-adherence over time. (c) Mean score on dietary adherence over time. (d) Mean score on stress reduction over time.

Figure 2a–d show the course of health behaviors over time. Univariate mixed models were constructed to assess the changes over time in the adherence-related measures. Overall, time showed significant, positive associations with adherence to exercise (*B* = 0.08, *p* < .001), dietary adherence (*B* = 0.20, *p* = .004), and stress reduction (*B* = 0.09, *p* = .033), indicating small but significant improvements in these behaviors over time (Table 2, Model 1). However, medication adherence decreased over time as indicated by a significant, positive association between medication non-adherence (*B* = 0.08, *p* < .001) and time. There were no significant improvements in adherence to medical advice or the probability of not smoking over time.

**Table 2:** Results From Multivariate Mixed Models for Adherence to Medical Advice and Health Behaviors

	Adherence to medical advice			Adherence to exercise			Medication adherence		
	Est.	*t*	*p*	Est.	*t*	*p*	Est.	*t*	*p*
Model 1 (unadj.)									
Time	0.02	1.38	.17	**0.08**	**3.81**	**<.001**	**0.08**	**4.08**	**<.001**
Model 3*									
Time	0.05	1.63	.10	**0.12**	**2.56**	**.01**	0.04	0.84	.40
Depressive sympt. × time	−0.00	−0.21	.84	−0.00	−0.42	.67	−0.00	−0.30	.76
Anxiety × time	−0.00	−0.32	.75	0.01	0.75	.45	−0.00	−0.22	.83
Optimism × time	−0.01	−0.74	.46	**−0.03**	**−2.20**	**.028**	0.00	0.33	.74
Pessimism × time	−0.01	−1.17	.24	0.01	1.26	.21	−0.00	−0.32	.75
Stress past year × time	0.02	1.15	.25	−0.02	−0.60	.55	0.03	1.23	.22
Resilience × time	**0.01**	**2.02**	**.044**	0.00	0.51	.61	0.00	1.03	.30
Type D personality × time	0.00	0.62	.54	−0.00	−1.82	.07	0.00	1.06	.29
Cardiac SE × time	0.00	1.48	.14	−0.00	−1.87	.06	0.00	0.59	.56
CR × time	−0.06	−1.61	.11	−0.08	−1.75	.08	0.05	0.99	.33
Model 4*									
Time	0.04	1.01	.31	0.08	1.48	.14	0.01	0.11	.91
Depressive sympt. × CR × time	0.02	1.92	.06	0.01	0.91	.37	0.01	0.56	.57
Anxiety × CR × time	−0.03	−2.20	.028	0.01	0.44	.66	−0.02	−1.02	.31
Optimism × CR × time	0.00	0.11	.92	0.02	0.73	.47	−0.01	−0.36	.72
Pessimism × CR × time	0.01	0.79	.43	0.01	0.47	.64	0.01	0.39	.70
Stress past year × CR × time	0.01	0.35	.73	0.02	0.42	.68	0.03	0.52	.60
Resilience × CR × time	0.00	0.61	.54	0.01	1.13	.26	0.01	0.55	.58
Type D personality × CR × time	0.00	1.13	.26	−0.00	−0.37	.71	0.00	1.19	.23
Cardiac SE × CR × time	0.00	0.70	.48	0.00	0.56	.58	0.01	1.36	.17
	Dietary adherence			Stress reduction			Smoking		
	Est.	*t*	p	Est.	t	p	Est.	t	p
Model 1 (unadj.)									
Time	**0.20**	**2.91**	**.004**	**0.09**	**2.14**	**.033**	0.01	0.48	.63
Model 3*									
Time	**0.44**	**2.96**	**.003**	0.14	1.57	.12	0.08	0.88	.38
Depressive sympt. × time	**−0.07**	**−3.02**	**.003**	−0.01	−0.96	.34	0.00	0.04	.97
Anxiety × time	**0.06**	**2.25**	**.024**	0.02	1.10	.27	−0.02	−1.24	.21
Optimism × time	**−0.09**	**−2.23**	**.026**	−0.03	−1.39	.17	0.02	0.94	.35
Pessimism × time	0.01	0.36	.72	0.01	0.68	.50	−0.01	−0.40	.69
Stress past year × time	−0.17	−1.84	.07	−0.01	−0.11	.91	0.04	0.73	.47
Resilience × time	−0.01	−0.66	.51	−0.02	−1.65	.10	−0.02	−1.47	.14
Type D personality × time	0.00	0.44	.66	−0.00	−0.52	.60	0.00	0.92	.36
Cardiac SE × time	−0.02	−1.96	.05	0.00	0.13	.89	0.01	1.08	.28
CR × time	**−0.34**	**−2.16**	**.031**	−0.15	−1.56	.11	0.06	0.56	.57
Model 4*									
Time	**0.33**	**1.98**	**.049**	0.11	1.08	.28	0.18	1.86	.06
Depressive sympt. × CR × time	−0.06	−1.27	.20	0.01	0.39	.70	−0.02	−0.70	.48
Anxiety × CR × time	0.07	1.19	.24	−0.00	−0.11	.92	−0.01	−0.36	.72
Optimism × CR × time	−0.06	−0.79	.43	0.09	1.84	.07	0.09	1.73	.08
Pessimism × CR × time	**−0.24**	**−3.62**	**<.001**	0.06	1.58	.11	−0.04	−1.01	.31
Stress past year × CR × time	0.07	0.37	.71	0.17	1.51	.13	**0.27**	**2.18**	**.029**
Resilience × CR × time	**0.08**	**2.34**	**.020**	−0.01	−0.48	.63	−0.01	−0.27	.79
Type D personality × CR × time	0.00	0.84	.40	0.00	0.87	.39	−0.01	−1.53	.13
Cardiac SE × CR × time	0.01	0.59	.56	−0.01	−1.31	.19	−0.02	−1.82	.07

^*^Adjusted for age, sex, educational level, civil status, previous cardiovascular events or procedures, comorbidities, genetic risk, PCI indication (elective vs. acute), obesity and hypertension. Bold values denote statistical significance at the *p* <0.05 level.

### Associations Between Psychological Factors, CR Participation, and Adherence

As expected, between-subject differences in psychological variables at baseline and adherence were found (Supplementary Table 2). Although these cross-sectional associations are important, the objective of the current study is to examine the association of psychological factors with *changes over time* in adherence to health behaviors.

### Associations Between Psychological Factors and Adherence Over Time

The results of the six separate, covariate-adjusted, mixed models demonstrated the associations between several psychological factors at baseline and the course of adherence to medical advice, exercise, and diet (Table 2, Model 3). No associations were found between any of the psychological factors or participation in CR and medication adherence, stress reduction, or smoking cessation.

#### Medical advice

**Fig. 3.**
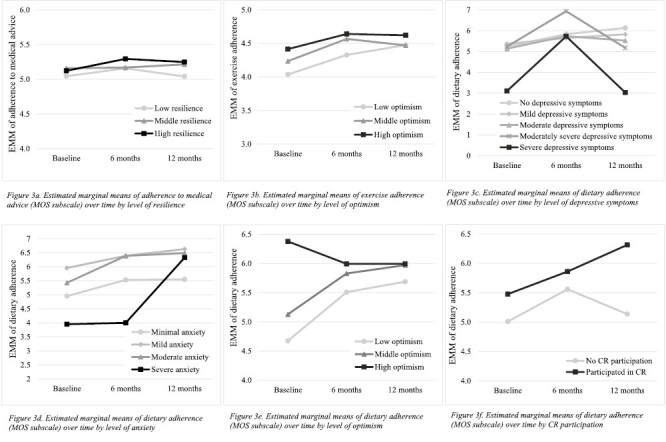
(a) Estimated marginal means of adherence to medical advice (Medical Outcomes Study [MOS] subscale) over time by level of resilience. (b) Estimated marginal means of exercise adherence (MOS subscale) over time by level of optimism. (c) Estimated marginal means of dietary adherence (MOS subscale) over time by level of depressive symptoms. (d) Estimated marginal means of dietary adherence (MOS subscale) over time by level of anxiety. (e) Estimated marginal means of dietary adherence (MOS subscale) over time by level of optimism. (f) Estimated marginal means of dietary adherence (MOS subscale) over time by CR participation.

A small association between resilience at baseline and the course of adherence to medical advice (*B* = 0.01, *p* = .044) was found, meaning the course of adherence to medical advice depended on the patients’ level of resilience (Fig. 3a). Patients who scored within the high range of resilience increased in adherence to medical advice from baseline to 6 months and then subsided, whereas for people who scored in the middle range of resilience, the average adherence to medical advice hardly changed. Patients characterized by low resilience showed an initial small improvement from baseline to 6 months, but then dropped back to baseline levels between 6 and 12 months.

#### Exercise

A small association between optimism at baseline and the course of exercise adherence (*B* = −0.03, *p* = .028) was found. Figure 3b demonstrates that exercise adherence increased from baseline to 12 months across the entire range of optimism, except for patients in the middle range of optimism, whose exercise adherence decreased over the second 6 months.

#### Dietary adherence

Low scores on depressive symptoms at baseline (*B* = −0.07, *p* = .003), anxiety at baseline (*B* = 0.06, *p* = .024), and optimism at baseline (*B* = −0.09, *p* = .026), and participation in CR (*B* = −0.34, *p* = .031) were all associated with improved dietary adherence over time. Figure 3c shows that for people with (moderately) severe depressive symptoms, dietary adherence improved from baseline to 6 months, but worsened from 6 to 12 months, whereas for patients who scored in the lower ranges of depressive symptoms, dietary adherence steadily increased. The reverse is shown for people with severe anxiety symptoms, as this group appeared to have improved most from 6 months to 12 months in dietary adherence, compared to people with all other levels of anxiety symptoms (Fig. 3d). Furthermore, for patients who scored in the low to middle levels of optimism, dietary adherence increased over time, whereas for patients who scored high on optimism, dietary adherence decreased from baseline to 6 months and remained this way until 12 months (Fig. 3e). Lastly, dietary adherence increased from baseline to 6 months for both patients who participated in CR and for patients who did not, but after 6 months, only the group who participated in CR continued to improve (Fig. 3f).

### Role of CR Participation in Associations Between Psychological Factors and Adherence Over Time

In model 4 (Table 2) the three-way interactions between CR, all psychological factors at baseline and time were examined for the six adherence outcomes. Results demonstrated a moderation effect of participation in CR on some of the associations between psychological factors at baseline and the 1-year course of adherence to medical advice, medication adherence, dietary adherence, and smoking, but not for exercise adherence and stress reduction.

#### Medical advice

**Fig. 4.**
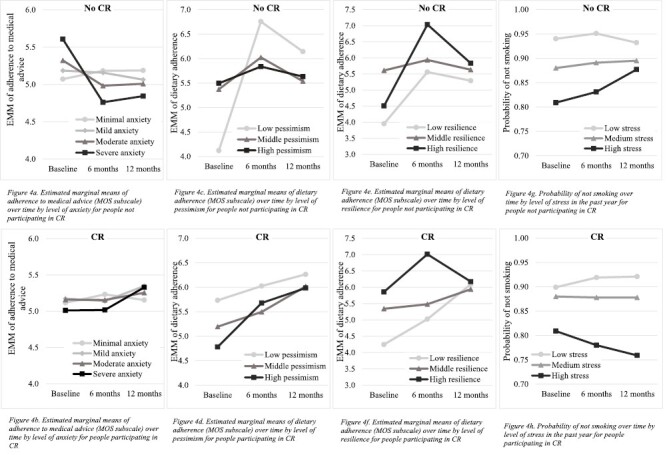
(a) Estimated marginal means of adherence to medical advice (MOS subscale) over time by level of anxiety for people not participating in cardiac rehabilitation (CR). (b) Estimated marginal means of adherence to medical advice (MOS subscale) over time by level of anxiety for people participating in CR. (c) Estimated marginal means of dietary adherence (MOS subscale) over time by level of pessimism for people not participating in CR. (d) Estimated marginal means of dietary adherence (MOS subscale) over time by level of pessimism for people participating in CR. (e) Estimated marginal means of dietary adherence (MOS subscale) over time by level of resilience for people not participating in CR. (f) Estimated marginal means of dietary adherence (MOS subscale) over time by level of resilience for people participating in CR. (g) Probability of not smoking over time by level of stress in the past year for people not participating in CR. (h) Probability of not smoking over time by level of stress in the past year for people participating in CR.

It was found that the relationship of anxiety at baseline (*B* = −0.03, *p* = .028) with the course of adherence to medical advice was moderated by CR. Figure 4a and b demonstrate that people with moderate to severe anxiety who did participate in CR improved in adherence to medical advice, whereas patients who scored in the moderate to severe range of anxiety who did not participate in CR decreased in adherence.

#### Dietary adherence

The relationships of pessimism at baseline (*B* = −0.24, *p* < .001) and resilience at baseline (*B* = 0.08, *p* = .020) with the course of dietary adherence were both moderated by participation in CR. Figure 4c and d show that for all patients who did not participate in CR, dietary adherence increased from baseline to 6 months and decreased from 6 months to 12 months, whereas it improved from baseline to 12 months for all patients participating in CR. This improvement was greatest for people who scored in the high range of pessimism. In terms of resilience, patients who scored in the low to middle ranges of resilience improved in both groups from baseline to 6 months, but this trend was only continued in the group that did participate in CR (Fig. 4e and f). The trajectory was similar for patients who scored high on resilience, regardless of CR participation.

#### Smoking

Analysis showed that CR participation moderated the associations of stress in the past year at baseline (*B* = 0.27, *p* = .029) with smoking prevalence. Figures 4g and h demonstrate that people who scored in the high range of stress in the past year and who did participate in CR decreased in probability of not smoking, whereas the people who scored similarly but did not participate in CR increased in probability of not smoking.

## Discussion

This study explored the role of psychological factors at baseline in the course of adherence to medical advice and to specific health behaviors. Additionally, it was investigated whether participation in CR moderated any of these associations. Our exploratory analysis demonstrated that several baseline psychological factors were associated with the 1-year course of adherence of all health behaviors, except stress reduction and smoking cessation. Participation in CR moderated some of these associations.

In general, adherence to exercise, dietary adherence, and stress reduction improved over time, with exercise and dietary adherence stabilizing after 6 months. Medication adherence was the only behavior that worsened over time. Smoking prevalence and adherence to medical advice remained stable. With respect to predictors, positive psychological factors in general were associated with overall better adherence, but not always with an upwards slope across time. Based on the results of this study, resilience, optimism, anxiety and depressive symptoms likely impact *patient capacity*, which in turn influences the balance between the *burden of treatment* and the *burden of illness*, following Shippee’s model (Fig. 1). This balance is further influenced through CR participation, that can be an effective tool to increase *patient capacity,* specifically for patients with high anxiety, high pessimism, and low resilience.

### Buffering Psychological Factors

Firstly, resilience was found to play an important role in adherence to medical advice, as patients who were lacking resilience were found to subside from 6 to 12 months post-PCI. Resilience has previously been shown to play a role in the *uptake* of health behavior [40]. The current study demonstrates its importance in *adherence* to medical advice as well, specifically from 6 to 12 months post-PCI.

Furthermore, both patients who scored low and patients who scored high on optimism had a more favorable course of exercise adherence than those with moderate levels of optimism. Previous research has predominantly demonstrated the role of high levels optimism in physical activity levels [41–43] and better CVD outcomes [44], but this association was mainly found in cross-sectional research. Previous short-term (2–12 weeks) prospective research on the relationship between optimism and physical activity showed optimism to have no relationship to physical activity over time [45–50], whereas some longer-term (6 months–6 years) prospective research did find a positive relationship between optimism and physical activity [41, 51–53], while others [25, 54] did not. The relationship between higher levels of optimism and physical activity appears time-variant and warrants further research. The association between lower optimism and exercise adherence found in the current study might be explained through the concept of defensive pessimism—a motivated cognitive strategy where people set low expectations to prepare for goal-relevant tasks and situations [55], which has previously been related to better physical performances [56]. However, this perspective requires further research.

Despite the positive role high optimism plays in exercise adherence, the results of this study also demonstrated that patients with high optimism have a less favorable course of dietary adherence. Previous research has shown both a cross-sectional relationship [43, 57] as well as a longitudinal relationship (18 weeks–6 years) [25, 26, 52, 54, 58], between high optimism and better dietary choices. However, previous research has also shown that optimism was not associated with dietary choices shortly after discharge (2 weeks) [47], indicating that the relationship between optimism and diet may vary over time, which is also demonstrated in the present study. This association may be due to unrealistic optimism (i.e., expecting a better personal future than is reasonably likely), which has previously been shown to be prevalent in patients with cardiovascular diseases [59, 60]. Due to the underestimation of future cardiovascular risk, patients may not feel inclined to adhere to dietary guidelines or may overestimate their current adherence to guidelines (response bias). The CR program in The Netherlands prioritizes exercise over diet, which may reduce unrealistic optimism about physical activity levels and might explain the difference in the associations between adherence to exercise and dietary behavior in patients with high levels of optimism. However, although unrealistic optimism and high trait optimism correlate, it is unclear whether the presence of unrealistic optimism can be derived from a high score on the LOT-R questionnaire, meaning we can only speculate about the presence of unrealistic optimism in this sample [46].

Cardiac self-efficacy was not found to be predictive of adherence to health behavior, despite being cross-sectionally related to health behaviors [61]. This could imply that, despite its role in generating motivation to change health behavior, its role in adhering to health behavior is less substantial than other psychological factors.

### Negative Chronic and Episodic Psychological Factors

Despite their roles in the uptake of health behavior, chronic psychological factors, that is, pessimism, stress in the past year, and Type D personality, were not found to play a role in adherence to health behavior. It is possible that, because of their more permanent nature, chronic negative psychological factors are more determinant of whether someone performs a behavior but are not predictive of relatively short-term changes in health behavior. The observed lack of association between personality factors and health behavior change is also found in previous research, suggesting that changes in personality factors are not strongly related to changes in health behaviors [62]. Conversely, negative episodic psychological factors were found to play a role in adherence behaviors, predominantly in dietary adherence. Patients with (moderately) severe depressive symptoms had a less favorable course of dietary adherence. This has previously been found in studies using a cross-sectional design, but the current study confirms the role depressive symptoms play across the 1-year adherence to dietary behavior [16].

Patients with high anxiety had a more favorable trajectory of dietary adherence. This is a surprising finding, as previous research has shown a relationship between high anxiety and less adherence to health behavior [63, 64]. The cross-sectional analysis of the EUROASPIRE study demonstrated that high levels of anxiety were associated with a reduced likelihood of making lifestyle changes [63]. However, a review on psychological factors in patients with heart failure concluded that the association of anxiety with both the level and course of adherence or self-care behaviors is inconsistent [65]. As the trajectory of adherence only improved from 6 to 12 months for patients scoring high on anxiety in our sample, it is possible that 6-month anxiety scores improved from baseline for this group, meaning their anxiety scores increased alongside their dietary adherence.

### Participation in CR

Participation in CR was only associated with the course of dietary adherence, with patients who participated in CR improving their dietary adherence from baseline to 12 months. The low uptake of and adherence to health behaviors in CR is a well-established problem in CHD patients [10, 11]. Our results demonstrate, however, that the success of participation in CR may depend on psychological characteristics. Participation in CR was shown to buffer the negative effects of high anxiety, high pessimism, and low resilience, emphasizing the importance of participation in CR for these patients specifically. This is an important finding, as anxiety and pessimism are prevalent in this patient population and have been shown to act as barriers to the uptake of health behavior [15,20,66]. It is therefore imperative to focus on including patients with a pessimistic or anxious disposition, as they may disproportionately benefit from CR. In earlier research, the positive effect of participation in CR on psychological factors, such as depression and anxiety, and overall mental health has already been established [67]. However, the resulting effect on adherence to health behaviors was thus far unexplored.

Despite this buffering role, participation in CR seemed to reverse the positive trajectory of participants with high stress in the past year in terms of the probability of not smoking. A possible explanation for this finding is that people who experience high levels of stress are more prone to experience stress when participating in CR. This could be due to some patients participating in CR despite experiencing barriers, such as a travel burden, gender disparity in CR sessions, or poor physical health, which might contribute to their elevated levels of psychological stress [68]. Another explanation could be that participants who were already motivated enough to quit smoking declined CR participation, meaning the group that did participate may have disproportionately consisted of patients who were less likely to quit smoking.

### Limitations and Strengths

The current study needs to be interpreted considering its limitations. The most important limitation is the collection of adherence measures through self-report, allowing for bias in these measurements [69, 70]. Future research could measure adherence through more objective measures, such as activity trackers, electronic pill bottles, food logs, or cotinine measurements. The present analyses did not correct for statistical Type 1 error related to multiple testing and therefore primarily provide an indication of the magnitude of the effect sizes of the associations between psychological factors and adherence to health behaviors. This comprehensive approach is a potential merit of this study as both psychological risk factors for CHD and adherence to relevant health behaviors for CHD are complicated constructs that require comprehensive analyses. However, the effect sizes of the statistically significant associations were often small and further replication studies are needed to investigate the robustness of the present observations. Furthermore, only participation in CR was considered, but not the number of sessions attended, intensity of participation, or location of CR, which might further moderate the associations between psychological factors and adherence. Psychological factors were only considered at baseline, as considering the psychological variables over time would overcomplicate the statistical models, which means the current study could not account for changes in psychological factors that might have increased our understanding of the role of psychological factors on adherence to health behaviors. Another limitation is that the present study only evaluated psychological factors with a known association with CHD outcomes and it is possible that other psychological factors are (more strongly) associated with adherence to health behaviors.

Strengths of the current study include the large sample size, which allowed for all psychological factors to be assessed in one model. Furthermore, due to the large sample size, we were able to statistically adjust for multiple common covariates in behavior change in patients with CHD.

### Clinical Recommendations

Screening for psychosocial risk factors for patients with CHD is in most cases limited to anxiety and depression [71]. However, the current study demonstrates the importance of considering other psychological risk factors, such as resilience, optimism, and pessimism when screening patients. Broadening the scope of psychosocial screening has been put forward before [16], and the ESC guidelines for cardiovascular prevention have advocated for screening for a broad set of risk factors since 2012 [72, 73]. Participation in CR could be emphasized for patients who score low on resilience, high on pessimism, and both high and low on optimism during screening specifically. Furthermore, previous studies have demonstrated the effectiveness of positive psychological interventions for CHD patients, which could be added to practice as well [74, 75], given the positive associations between positive psychological factors and adherence to health behaviors. It is recommended that these interventions focus specifically on strengthening resilience, for example, by focusing on the social support available to participants [76], to improve adherence to medical advice, and on cultivating realistic optimism to improve adherence to exercise and potentially adherence to a healthy diet. Lastly, as adherence to health behaviors either does not improve or decreases after the 6-month follow-up, it could be worthwhile to invite patients to a “refresher” session addressing optimal health behaviors and mental well-being after a couple of months.

### Future Research

Future research could examine what predicts adherence to multiple behaviors. Previous research has already demonstrated high correlations between adherence to different behaviors (healthy adherer effect) [77], but the role of psychological factors in these high concurrencies has not been investigated. As the current study showed that a positive association of a psychological factor with adherence to one behavior, can coincide with a negative association of the same psychological factor with adherence to a different behavior (i.e., high optimism), it is important to investigate psychological factors in the context of multiple behaviors. Furthermore, future research could measure psychological factors over time as well, to account for possible changes over time and the potential implications these changes have for adherence to health behaviors. Since the current study did not consider knowledge about health behavior as a potential covariate, despite research showing its importance in changing health behavior, future studies could take this factor into account [78]. Future research could additionally assess what type of smoking patients engaged in (e.g., the use of e-cigarettes).

In conclusion, the current study demonstrated the role of psychological factors in adherence to health behaviors, and specifically in adherence to dietary guidelines. Both positive and negative psychological factors are associated with adherence to health behaviors and need to be considered in clinical settings. Participation in CR was shown to buffer against the negative effects of high anxiety, high pessimism, and low resilience. However, participation in CR also seemed related to an increased probability of smoking in patients with high stress in the past year. Future research could investigate the role of psychological factors in adherence to multiple health behaviors.

## Supplementary Material

kaae008_suppl_Supplementary_Material
