# Supplementary material for: Associations Between Psychological Factors and Adherence to Health Behaviors After Percutaneous Coronary Intervention: The Role of Cardiac Rehabilitation
Source: Ann Behav Med. 2024 Mar 2;58(5):328–40. doi: 10.1093/abm/kaae008 (PMC11008588; doi:10.1093/abm/kaae008)
Supplement: kaae008_suppl_Supplementary_Material [file kaae008_suppl_supplementary_material.docx]

Electronic Supplemental Table 1. Results from multivariate mixed models for adherence to medical advice and health behaviors (negative affectivity, social inhibition, NA*NA, SI*SI)

|  | *Adherence to medical advice* | | | *Adherence to exercise* | | | *Medication adherence* | | | *Dietary adherence* | | | *Stress reduction* | | | *Smoking* | | | |
| --- | --- | --- | --- | --- | --- | --- | --- | --- | --- | --- | --- | --- | --- | --- | --- | --- | --- | --- | --- |
|  | Est. | *t* | *p* | Est. | *t* | *p* | Est. | *t* | *p* | Est. | *t* | *p* | Est. | *t* | *p* | Est. | *t* | *p* |  |
| **Model 2*** |  |  |  |  |  |  |  |  |  |  |  |  |  |  |  |  |  |  |  |
| Negative affectivity (NA) | .01 | .83 | .41 | **.03** | **2.29** | **.02** | -.01 | -1.19 | .23 | .01 | .14 | .89 | .03 | 1.32 | .19 | .00 | .08 | .93 |  |
| Social inhibition (SI) | .01 | 1.66 | .10 | .01 | .94 | .35 | -.00 | -.05 | .96 | .01 | .43 | .67 | .00 | .19 | .85 | .05 | 1.47 | .14 |  |
| NA × NA | .00 | 1.34 | .18 | **.002** | **2.19** | **.029** | -.00 | -.51 | .61 | .00 | .58 | .56 | -.00 | -.62 | .54 | .00 | -.18 | .86 |  |
| SI × SI | .00 | 1.12 | .26 | .00 | 1.54 | .12 | .00 | .55 | .58 | .00 | .47 | .64 | -.00 | -.41 | .68 | .00 | .80 | .42 |  |
| **Model 3*** |  |  |  |  |  |  |  |  |  |  |  |  |  |  |  |  |  |  |  |
| NA × time | -.01 | -.71 | .48 | .01 | 1.21 | .23 | -.01 | -.92 | .36 | -.02 | -.51 | .61 | .00 | .18 | .86 | -.01 | -.59 | .55 |  |
| SI × time | -.00 | -.63 | .53 | .01 | 1.78 | .08 | -.01 | -.90 | .37 | -.02 | -.87 | .39 | -.01 | -.50 | .62 | -.01 | -.58 | .56 |  |
| NA × NA × time | -.00 | -.52 | .60 | -.00 | -.87 | .39 | .00 | .57 | .57 | **-.004** | **-1.98** | **.048** | .00 | .46 | .65 | -.00 | -.73 | .47 |  |
| SI × SI × time | -.00 | -.56 | .58 | .00 | .02 | .99 | .00 | 1.03 | .31 | .00 | .40 | .69 | .00 | .61 | .54 | -.00 | -.86 | .39 |  |
| **Model 4*** |  |  |  |  |  |  |  |  |  |  |  |  |  |  |  |  |  |  |  |
| NA × CR × time | -.00 | -.16 | .87 | .02 | .89 | .37 | -.00 | -.21 | .83 | -.07 | -1.03 | .30 | .00 | .07 | .95 | .08 | 1.75 | .08 |  |
| SI × CR × time | -.02 | -1.61 | .11 | .01 | .86 | .39 | -.02 | -1.37 | .17 | -.02 | -.49 | .63 | .00 | .09 | .93 | .01 | .27 | .78 |  |
| NA × NA × CR × time | **-.003** | **-2.85** | **.004** | -.00 | -1.85 | .06 | **-.003** | **-2.53** | **.012** | -.01 | -1.33 | .18 | .00 | 1.09 | .28 | .00 | -.18 | .86 |  |
| SI × SI × CR × time | .00 | .90 | .37 | -.00 | -.54 | .59 | .00 | .25 | .80 | .00 | .09 | .93 | -.00 | -1.92 | .06 | .01 | 3.11 | .002 |  |

* adjusted for age, sex, educational level, civil status, previous cardiovascular events or procedures, comorbidities, genetic risk, PCI indication (elective vs. acute), obesity and hypertension.

Electronic Supplemental Figure 1. Estimated marginal means of dietary adherence (MOS subscale) over time by level of NA*NA

Electronic Supplemental Figure 4. Estimated marginal means of medication non-adherence over time by level of NA*NA for people not participating in CR

Electronic Supplemental Figure 6. Probability of not smoking over time by level of SI*SI for people not participating in CR

Electronic Supplemental Figure 5. Estimated marginal means of medication non-adherence over time by level of NA*NA for people participating in CR

Electronic Supplemental Figure 7. Probability of not smoking over time by level of SI*SI for people participating in CR

Electronic Supplemental Figure 3. Estimated marginal means of adherence to medical advice (MOS subscale) over time by level of NA*NA for people participating in CR

Electronic Supplemental Figure 2. Estimated marginal means of adherence to medical advice (MOS subscale) over time by level of NA*NA for people not participating in CR

*Electronic Supplemental Table 2. Associations between psychological factors and adherence to health behaviors*

|  | *Medical advice* | | | *Adherence to exercise* | | | *Medication adherence* | | | *Dietary adherence* | | | *Stress reduction* | | | *Smoking* | | |
| --- | --- | --- | --- | --- | --- | --- | --- | --- | --- | --- | --- | --- | --- | --- | --- | --- | --- | --- |
|  | Est. | *t* | *p* | Est. | *t* | *p* | Est. | *t* | *p* | Est. | *t* | *p* | Est. | *t* | *p* | Est. | *t* | *p* |
| **Model 2*** |  |  |  |  |  |  |  |  |  |  |  |  |  |  |  |  |  |  |
| Time | .02 | 1.00 | .32 | **.08** | **3.32** | **<.001** | **.09** | **4.06** | **<.001** | **.22** | **2.96** | **.003** | **.14** | **3.22** | **<.001** | .02 | .40 | .69 |
| Depressive sympt. | -.01 | -1.52 | .13 | **-.03** | **-3.56** | **<.001** | .00 | .32 | .75 | .06 | 1.89 | .06 | .01 | .65 | .52 | -.01 | -.36 | .72 |
| Anxiety | -.00 | -.65 | .51 | .00 | .25 | .80 | .00 | .51 | .61 | -.01 | -.18 | .86 | .02 | 1.12 | .26 | .03 | 1.04 | .30 |
| Optimism | **.02** | **2.19** | **.029** | **.05** | **2.92** | **.004** | -.02 | -1.28 | .20 | **.11** | **2.09** | **.037** | **.08** | **2.66** | **.008** | .07 | 1.53 | .13 |
| Pessimism | **.04** | **4.26** | **<.001** | .02 | 1.57 | .12 | -.00 | -.26 | .79 | -.01 | -.17 | .87 | -.03 | -1.07 | .28 | .06 | 1.39 | .16 |
| Stress past year | .01 | .48 | .63 | .06 | 1.58 | .11 | .06 | 1.74 | .08 | **.31** | **2.42** | **.016** | **.31** | **4.47** | **<.001** | .06 | .45 | .65 |
| Resilience | .01 | 1.82 | .07 | **.02** | **3.23** | **.001** | .00 | .23 | .82 | .03 | 1.58 | .12 | **.06** | **4.76** | **<.001** | **.05** | **2.21** | **.027** |
| Type D personality | -.00 | -1.85 | .07 | **-.002** | **-2.11** | **.036** | .00 | 1.29 | .20 | -.00 | -.67 | .50 | -.00 | -.33 | .74 | -.00 | -.55 | .58 |
| Cardiac SE | **.01** | **6.48** | **<.001** | **.02** | **6.92** | **<.001** | .00 | -1.60 | .11 | .00 | .01 | 1.00 | -.00 | -.15 | .88 | **.04** | **4.18** | **<.001** |
| CR | -.08 | -1.89 | .06 | -.04 | .60 | .55 | .03 | .49 | .63 | **-.52** | **-2.35** | **.019** | -.15 | -1.20 | .23 | .13 | .58 | .57 |

* adjusted for age, sex, educational level, civil status, previous cardiovascular events or procedures, comorbidities, genetic risk, PCI indication (elective vs. acute), obesity and hypertension.
